# Cognitive complaints in cancer survivors and expectations for support: Results from a web–based survey

**DOI:** 10.1002/cam4.2069

**Published:** 2019-03-18

**Authors:** Marie Lange, Idlir Licaj, Bénédicte Clarisse, Xavier Humbert, Jean‐Michel Grellard, Laure Tron, Florence Joly

**Affiliations:** ^1^ Clinical Research Department Centre François Baclesse Caen France; ^2^ Normandie Univ, UNICAEN, INSERM, ANTICIPE Caen France; ^3^ Cancer and Cognition Platform Ligue Nationale Contre le Cancer Caen France; ^4^ Department of General Medicine, Medical School Caen France; ^5^ Department of Pharmacology University Hospital of Caen Caen France; ^6^ Normandie Univ, UNICAEN Caen France; ^7^ University Hospital of Caen Caen France

**Keywords:** cancer treatments, chemobrain, cognitive complaints, survey

## Abstract

**Background:**

Cognitive complaints are common in cancer survivors. We aimed to assess cognitive complaints in cancer survivors and the associated factors using a large web–based survey.

**Methods:**

This online survey was proposed to cancer survivors. Participants completed several questions on cognitive complaints experience, expectations for support of cognitive difficulties, preexisting knowledge about chemotherapy–associated cognitive problems and demographic and medical variables. We used multivariable logistic regression models to estimate Odds Ratios and 95% confidence intervals to estimate associations.

**Results:**

Among 1610 eligible participants (median age 52 [21‐84]), >85% (n = 1393) were breast cancer survivors. Median postcancer treatment time (excluding hormone therapy) was 2.83 years [0.8‐33]. Seventy five percent of the participants (n = 1214) reported cognitive complaints related to cancer treatments. Cognitive difficulties had an impact on work resumption for 76% of the participants (n = 754/982). Most cancer survivors would like to receive support (75%, n = 909) and especially cognitive training (72%, n = 658). Chemotherapy was strongly associated with cognitive complaints (multivariable OR = 3.67, 95% CI: 2.80‐4.82). Self–reported sleep difficulties (OR_often vs. never_ = 2.84, 95% CI: 1.80‐4.47), preexisting knowledge about chemotherapy–associated cognitive problems (OR_No vs. Yes_ = 1.69, 95% CI: 1‐29‐2.22) and age (OR_21‐64 vs. ≥65_ = 0.37, 95% CI: 0.23‐0.58) were also associated with cancer–related cognitive complaints.

**Conclusions:**

According to this large web–based survey including mainly breast cancer survivors, cognitive complaints were reported by three quarters of participants, which reinforces that cognitive difficulties are a major issue in cancer survivors. Chemotherapy, self–reported sleep difficulties and preexisting knowledge about chemotherapy–associated cognitive problems were strongly associated with cancer–related cognitive complaints. Most cancer survivors wished to receive support and especially cognitive training.

## 1 BACKGROUND

Memory and attention complaints are common in cancer survivors even several years after treatments, mainly after chemotherapy or hormonal therapy.^1^ These difficulties can have a negative impact on patients’ quality of life and disturb ability to work.^2^, ^3^, ^4^

Patients with cognitive complaints do not systematically have objective cognitive impairment assessed within neuropsychological tests,^5^ but these two measures are complementary. Cognitive complaints, strongly related with psychological factors,^6^, ^7^ are important to identify and take into account due the significant impact they could have on the quality of life.

Even if these difficulties seem to be a transient problem, some cancer patients could have difficulties in the long run. Recent studies that have assessed cognitive complaints in cancer patient cohorts, principally in women with breast cancer, showed that on average 5‐7 years post diagnosis, 46 to 60% of the survivors had cognitive complaints.^8^, ^9^, ^10^ Nevertheless, six months after chemotherapy, results of breast cancer patients suggested partial recovery from cognitive complaints but without returning to pretreatment level.^11^ Among older breast cancer survivors followed annually for 7 years, half of the patients followed a phase shift trajectory (this subgroup of patients had more cognitive complaints than older adults with no cancer history but followed a parallel trajectory to older adults with no cancer history) and a small subset presented accelerated cognitive decline.^8^

Studies on patients’ expectations for support of cognitive disorders are scarce. Some qualitative study or small survey reported that breast cancer patients (n = 13) would like support to address their cognitive difficulties^12^ and would be interested in cognitive support strategies such as cognitive training.^13^ However, it was not explored which approach the patients would like to cope with cognitive difficulties among cognitive, physical or therapeutic interventions.

In the present study, we assessed the frequency of cognitive complaints in cancer survivors and the association with various demographic and cancer–related factors. We further described the impact of cognitive difficulties on work resumption and explored participants’ expectations for support of cognitive disorders.

## 2 METHODS

The study was a web‐based cross–sectional survey.

### 2.1 Procedure/network

Invitation to register to the network “*Seintinelles” *was displayed in the five biggest cancer centers through the French territory and in the main university hospitals of the largest cities in France: Paris, Marseille, Lyon, Bordeaux, Nice, Lille, and Toulouse. In addition, advertisement inviting cancer patients to register to the network were also displayed in large public media and social media continuously starting from 2014. Furthermore, all network registered participants (14 677 in September 2016) were invited via email to complete a web–based questionnaire to assess cancer posttreatment cognitive complaints. The participation rate was 38.30%.

Participants were recruited through the national cancer patients network, named *Seintinelles* platform,^14^ from 10‐2016 to 10‐2017.

The average time to complete the online survey was 30 minutes. All participants gave their consent on the survey website. The study was approved by the French Advisory Committee on treatment of research information and the Data Protection Agency (authorization request N°916059).

### 2.2 Participants

Eligible participants were any cancer patients, except primary brain cancer and brain metastasis, aged ≥18 years, French residents, who have finished their curative treatments (chemotherapy and/or radiotherapy; participation was possible if hormone therapy was ongoing), and with no history of progressive neurological or psychiatric disease nor drug abuse.

### 2.3 Assessment

Cognitive complaints experienced were firstly assessed through the question: “Have you (had) memory, concentration, finding words or other cognitive difficulties during or after cancer treatments?” For participants who responded “Yes”, additional questions included: start of cognitive difficulties, duration of difficulties, impact on work resumption, preexisting knowledge about chemotherapy–associated cognitive problems, previous participation in cognitive study or cognitive support, and expectations for support (through a list of proposals).

Demographic variables included gender, age, education, marital, and employment status. Cancer–related variables included type of cancer, metastatic status, cancer treatments, time since completing curative treatment (chemotherapy ± radiotherapy), time since completing hormone therapy, and symptoms following cancer treatments (among a list of symptoms). Other variables included menopausal status, body mass index (BMI), comorbidity with treatment, history of nonprogressive neurological diseases, self–reported sleep difficulties and physical activity. Psychotropic treatments were assessed in three steps: first step survivors were asked “Do you take drugs like sleeping pills, antidepressants, anxiolytics, or neuroleptics?”. For those who answered “yes”, they pursued with two additional steps: they were asked to specify the name of the drug and the frequency of intake.

### 2.4 Statistical analysis

For the distribution of selected characteristics of the study population, we used percentages or means with standard deviations. Characteristics of the study population associated with cancer–related cognitive complaints were assessed using bilateral chi‐squared tests for categorical variables and one‐way ANOVA or Kruskal‐Wallis tests for continuous variables.

The four most common cancer treatments, chemotherapy, radiotherapy, hormone therapy and surgery were coded as binary variables (yes, no). Patients who received chemotherapy either alone or in combination with other cancer treatments were coded “Yes” and those who did not receive chemotherapy (alone or in combination) were coded “No”.

Logistic regression models were built as recommended by Veierød et al.^15^ We performed univariable logistic models for each covariate to assess the potential association with cancer–related cognitive complaints and included the covariates significant at 20% level in a multivariable logistic regression model.^16^ Finally, the most parsimonious model was selected using a stepwise algorithm based on the Bayesian information criteria and included the following variables: postcancer curative treatment time (≤1, 1‐3, and ≥3 years), metastatic status (yes, no), age (21‐64, ≥65 years), employment status (full‐time or part‐time, sick leave, student or retired, unemployment and other), self–reported sleep difficulties (never, sometimes, often), and the frequency of psychotropic treatments were measured by asking the frequency of use and the specific names of the drugs used. (never, <1/month, ≥1/month and <1/week and ≥1/week), preexisting knowledge about chemotherapy–associated cognitive problems (yes, no). Similar multivariable models were estimated binary for each treatment.

We conducted several sensitivity analyses: an exclusive cancer treatments variable was created by categorizing the main possible cancer treatment combinations based on breast cancer patients which represents >85% of cancer survivors in our survey. Further multivariable analyses were repeated among breast cancer survivors exclusively.

All statistical analyses were performed using STATA version 14 (Stata Corp, College Station, TX).

## 3 RESULTS

**Figure 1:**
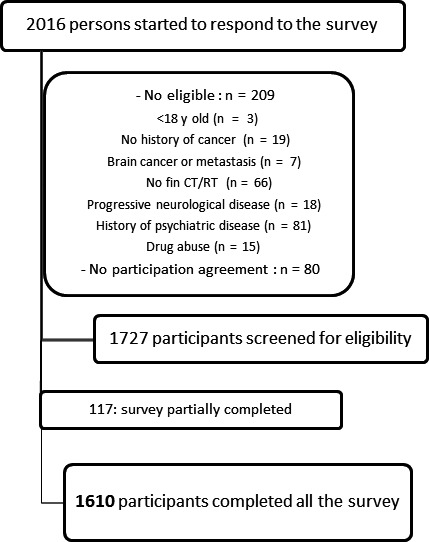
Flow chart

Among the 2016 persons who started to respond to the survey, 1727 gave their consent and were considered eligible and 1610 have completed all the survey (Figure 1). Participants’ characteristics are described in the Table 1. The median age was 52 [21‐84], 98% (n = 1579) of the participants were women, 70% (n = 1124) had high education level and >85% (n = 1393) were breast cancer survivors. Most of the participants had localized cancers (87%, n = 1404), had surgery (87%, n = 1480) and received radiotherapy (84%, n = 1356), and/or chemotherapy (77%, n = 1233) and/or hormone therapy (66%, n = 1059). Median postcancer curative treatment time (excluding hormone therapy) was 2.87 years [0.8‐33]. Five percent of the participants (n = 82) had a history of nonprogressive neurological disease (mainly head trauma with loss of consciousness), 17% (n = 273) had a weekly consumption of psychotropic medications and 58% (n = 927) reported to have often self–reported sleep difficulties.

**Table 1:** Demographic, clinical and lifestyle characteristics of participants

**Demographic**	All sample (n = 1610)	No cancer cognitive complaints experience, n = 396 (25%)	Cancer cognitive complaints experience, n = 1214 (75%)	*P* (χ^2^ or Student Exact Fisher or Kruskal Wallis)
Female (n, %)	1579 (98)	380 (96)	1199 (99)	<0.001
Age (y) mean (SD) [range 21‐84]	51.8 (10)	55.39 (11)	50.65 (9)	<0.001
21‐64 (%)	1440 (89)	305 (77)	1135 (93)	
≥65 (%)	170 (11)	91 (23)	79 (7)	<0.001
Education level (n, %), low	58 (4)	16 (4)	42 (3)	0.258
Middle	428 (27)	93 (23)	335 (28)	
High	1124 (70)	287 (72)	837 (69)	
Employment (n,%), full‐time or part‐time	882 (55)	196 (49)	686 (57)	0.015
Sick leave	230 (14)	26 (7)	151 (12)	
Student, retired	310 (19)	129 (33)	181 (15)	
Unemployment	75 (5)	14 (3)	61 (5)	
Other	113 (7)	31 (8)	135 (11)	
Married/partnered (n, %)	1174 (73)	279 (71)	895 (74)	0.204
*Clinical*				
Cancer (n, %): breast	1393 (87)	327 (83)	1066 (88)	
Gynecological	51 (3)	18 (5)	33 (3)	
Hematologic	51 (3)	15 (4)	36 (3)	
Other	115 (7)	36 (9)	79 (7)	0.057
Localized cancer (n, %)	1404 (87)	361 (91)	1043 (86)	0.007
Cancer treatments (n, %)				
Surgery	1480 (87)	353 (89)	1127 (93)	0.019
Chemotherapy	1233 (77)	220 (56)	1013 (83)	<0.001
Radiotherapy	1356 (84)	309 (78)	1047 (86)	<0.001
Hormone therapy	1059 (66)	221 (56)	838 (69)	<0.001
Targeted therapy	143 (9)	30 (8)	113 (9)	0.293
Other	101 (6)	22 (6)	79 (7)	0.498
Median postcancer curative treatment time^a^ (y) [range]	2.87 [0.08‐33]	3.00 [0.08‐33]	2.50 [0.08‐32]	<0.001
Median time posthormone therapy (n = 1320) (y) [range]	2.00 [0.08‐20]	3.00 [0.08‐20]	2.00 [0.08‐17]	0.005
Having at least one symptom postcancer treatments	1349 (84)	238 (60)	1111 (92)	<0.001
BMI kg/m^2^ (SD)	24.62 (6)	24.34 (4)	24.70 (6)	0.2751
Postmenopausal status (%) (n = 1579)	1124 (71)	278 (73)	846 (71)	0.330
Comorbidity with treatment (n, %)^b^	467 (29)	129 (33)	338 (28)	0.071
Disease of circulatory system	202 (43)	63 (49)	139 (41)	0.133
Endocrine, nutritional and metabolic diseases	198 (42)	56 (43)	142 (42)	0.784
Other	48 (10)	17 (13)	31 (9)	0.239
History of nonprogressive neurological disease (n, %)	82 (5)	27 (7)	55 (5)	0.072
Frequency of psychotropic treatments (n, %)				
Never	1058 (66)	286 (72)	772 (64)	
<1/month	198 (12)	42 (11)	156 (13)	
≥1/month and <1/wk	81 (5)	18 (5)	63 (5)	
≥1/wk	273 (17)	50 (13)	223 (18)	0.014
Self–reported sleep difficulties (%)				
Never	114 (7)	45 (11)	69 (6)	
Sometimes	569 (35)	170 (43)	399 (33)	
Often	927 (58)	181 (46)	746 (61)	<0.001
*Physical activity*				
Physical activity (n, %)				
None or monthly	517 (32)	131 (33)	386 (32)	
1/wk	309 (19)	76 (19)	233 (19)	
2/wk	407 (26)	86 (22)	321 (26)	
≥3/wk	377 (23)	103 (26)	274 (23)	0.231
Preexisting knowledge about chemotherapy–associated cognitive problems				
Preexisting knowledge (n, %)	618 (38)	102 (26)	516 (42)	<0.001

a Excluding hormone therapy.

b Participants may have more than one comorbidity.

Eighty four percent of the survivors (n = 1389) had listed posttreatment symptoms. Among these survivors, fatigue (75%, n = 1036), cognitive difficulties (62%, n = 856) and pain (42%, n = 578) were the most troublesome.

Thirty eight percent (n = 618) had preexisting knowledge about chemotherapy–associated cognitive problems (Table 1). Before this survey, 3% (n = 43) of the survivors participated to cognitive study or had cognitive support. Among these 44% (n = 19) had a neuropsychological assessment or neurological consultation, 40% (n = 17) received cognitive training and 16% (n = 7) had other support (such as physical activity or psychological support) or participated to cognitive study outside the oncology field.

### 3.1 Cognitive complaints

Seventy five percent of the participants (n = 1214) had cognitive complaints related to cancer treatments (Table 2). Cancer–related cognitive complaints started mainly during chemotherapy (35%, n = 419), after chemotherapy (30%, n = 360) and during hormone therapy (15%, n = 186) and lasted a median of 2 years [0.08‐32].

**Table 2:** Characteristics of cancer cognitive complaints

	n (%)
Cancer cognitive complaints (/1610)	1214 (75)
Start of cancer cognitive complaints (n = 1214)	
Before all cancer treatment	22 (2)
After surgery and before adjuvant treatment	47 (4)
During radiotherapy	24 (2)
During chemotherapy	419 (35)
During hormone therapy	186 (15)
After radiotherapy	84 (7)
After chemotherapy	360 (30)
After hormone therapy	30 (2)
Other	42 (3)
Median during time of cancer cognitive complaints (n = 1214) (y) [range]	2 [0.08‐32]
Impact on resumption of work (n=982^a^)	
None	79 (8)
Not really	149 (15)
A little	367 (37)
A lot	387 (39)

a Not concerned, n = 232.

Cognitive difficulties were reported to have an impact on work resumption in 76% of the participants (39%, n = 387 important impact and 37%, n = 367, little impact).

**Figure 2:**
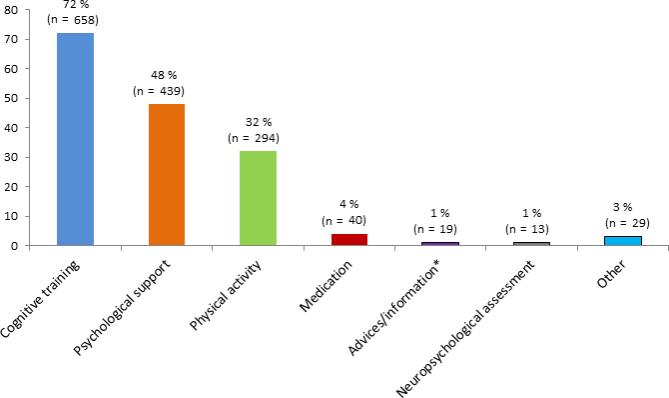
Expectations for support of cognitive complaints among n = 909 participants with cognitive complaints who were interested in cognitive support.

Among survivors who had cancer–related cognitive complaints, 75% (n = 909) would benefit from support of their cognitive difficulties. More frequent expectations for support (Figure 2) were cognitive training (72%, n = 658), psychological support (48%, n = 439) and physical activity (32%, n = 294).

### 3.2 Relations between cancer–related cognitive complaints and other variables

Table 1 shows that compared to participants who had no cancer–related cognitive complaints, participants who had cancer–related cognitive complaints were more often women, were younger, received more often chemotherapy, radiotherapy and hormone therapy, were more likely to have self–reported sleep difficulties and have more preexisting knowledge about chemotherapy–associated cognitive problems (*P* < 0.001). They also had more extensive cancer at diagnosis (*P* = 0.007). Participants reporting cancer–related cognitive complaints were more likely to be employed (full‐time or part‐time) (*P* = 0.015). Among employed participants, participants who had cancer–related cognitive complaints were more likely to work part‐time than full‐time (*P* = 0.005, data not shown).

Education level, marital status, BMI, menopausal status, comorbidities with treatment, history of nonprogressive neurological disease and frequency of physical activity were not significantly associated to cancer–related cognitive complaints.

### 3.3 Factors associated with duration of cognitive complaints

In participants who had cancer–related cognitive complaints, the duration of cognitive complaints was associated with employment status (cognitive complaints lasted less time among participants on sick leave, *P* < 0.001) and physical activity (participants who practiced more physical activity had shorter cognitive complaints, *P* < 0.001, Table 3). Age, education level, marital status, self–reported sleep difficulties, and preexisting knowledge about chemotherapy–associated cognitive problems were not associated with duration of cancer–related cognitive complaints.

**Table 3:** Factors associated with duration of cognitive complaints in years, among participants with cancer–related cognitive complaints

n = 1213	Mean (SD)	*P* Kruskal Wallis
Age in years		
21‐64	3.1 (2.9)	
≥65	3.6 (3.2)	0.83
Education level		
Low	3.6 (3.6)	
Middle	3.2 (2.8)	
High	3.1 (2.9)	0.18
Employment		
Full‐time or part‐time	3.2 (2.7)	
Sick leave	1.7 (1.9)	
Student, retired	3.8 (4.1)	<0.001
Unemployment	3.5 (2.5)	
Other	3.4 (3.4)	
Married/partnered		
No	3.3 (2.9)	0.16
Yes	3.1 (3.0)	
Self–reported sleep difficulties		
Never	2.8 (3.1)	
Sometimes	3.1 (3.3)	0.21
Often	3.1 (2.8)	
Physical activity		
None or <once a week	3.4 (3.0)	
Once a week	3.2 (2.9)	<0.001
Twice a week	2.9 (2.8)	
≥3 times a week	2.9 (3.3)	
Preexisting knowledge about chemotherapy–associated cognitive problems		
No	3.2 (3.0)	0.37
Yes	3.0 (2.9)	

### 3.4 Factors associated with cancer–related cognitive complaints

Results of multivariable analyses are presented in Table 4.

**Table 4:** Multivariable odds ratios (OR) and 95% confidence intervals (CI) of cancer cognitive complaints (n = 1610) and clinical, demographic and lifestyle characteristics based on chemotherapy model

	Cancer cognitive complaints		
	n cases 1214	Model	
		Multivariable^b^	
		OR	95% CI
Chemotherapy			
No	201	1.00	Reference
Yes	1013	**3.67**	**2.80‐4.82**
Postcancer curative treatment time			
≤1 y	342	1.00	Reference
1‐3 y	449	1.04	0.73‐1.45
≥3 y	423	0.72	0.52‐1.01
Cancer without metastasis			
No	171	1.00	Reference
Yes	1043	1.21	0.80‐1.84
Age			
21‐64 y	1135	1.00	Reference
≥65 y	79	**0.37**	**0.23‐0.58**
Employment status			
Employed (full‐time or part‐time)	686	1.00	Reference
Sick leave	151	1.27	0.78‐2.07
Student, retired	181	0.83	0.56‐1.24
Unemployment	61	1.10	0.58‐2.06
Other	135	1.12	0.72‐1.76
Self–reported sleep difficulties			
Never	69	1.00	Reference
Sometimes	399	**1.67**	**1.06‐2.61**
Often	746	**2.84**	**1.80‐4.47**
Frequency of psychotropic treatments			
Never	772	1.00	Reference
<1/month	156	1.32	0.88‐1.97
≥1/month and <1x/wk	63	1.23	0.67‐2.56
≥1/wk	223	1.27	0.88‐1.84
Preexisting knowledge^a^			
No	698	1.00	Reference
Yes	516	**1.69**	**1.29‐2.22**

In bold in the table: significant results.

a Preexisting knowledge about chemotherapy–associated cognitive problems.

b Mutually adjusted.

Chemotherapy was strongly associated with cancer–related cognitive complaints (Table 4, multivariable analyses; OR = 3.67, 95% CI: 2.80‐4.82). Self–reported sleep difficulties (OR_often vs. never_ = 2.84, 95% CI: 1.80‐4.47), preexisting knowledge about chemotherapy–associated cognitive problems (OR_No vs. Yes_ = 1.69, 95% CI: 1‐29‐2.22) and age (OR_21‐64 vs. ≥65_ = 0.37, 95% CI: 0.23‐0.58) were also associated with cancer–related cognitive complaints.

Other cancer treatments, namely hormone therapy (multivariable OR = 1.65, 95% CI: 1.29‐2.11) and radiotherapy (multivariable OR = 1.70, 95% CI: 1.24‐2.30) had a significant impact on cancer–related cognitive complaints (data not shown).

The odds of cancer cognitive complaint were reduced when comparing survivors who got (i) surgery and radiotherapy, or (ii) surgery, radiotherapy and hormonotherapy or (iii) surgery and targeted therapy when compared to those who went through surgery, radiotherapy and chemotherapy (reference group). In contrast, the odds of developing cancer cognitive complaints were increased for survivors who went through surgery, radiotherapy, chemotherapy, and hormonotherapy while compared to surgery, radiotherapy and chemotherapy (SA Table 1). When we restricted the analyses to breast cancer survivors only, similar results were observed (SA Table2).

## 4 DISCUSSION

Using a large web–based survey, we observed that a large proportion of cancer survivors report cognitive complaints and associate these with their cancer treatments. Chemotherapy was strongly associated with cognitive complaints. Self–reported sleep difficulties and preexisting knowledge about chemotherapy–associated cognitive problems were also strongly associated with cancer–related cognitive complaints. Cognitive difficulties had an impact on work resumption for 76% of the participants who had cancer–related cognitive complaints. Most cancer survivors wished to receive support and especially cognitive training.

In the present study, 75% of the participants had cancer–related cognitive complaints. This important rate may partly result from the procedures for participation, on a voluntary basis of subjects already registered in a network dedicated to cancer, and historically breast cancer. However, it remains consistent with literature. Indeed, recent studies in cancer patient cohorts, principally in breast cancer women, have shown that 46 to 60% of the survivors had cognitive complaints^8^, ^9^, ^10^ and a literature review has reported that up to 75% of the cancer patients experience cognitive difficulties,^17^ which reinforces that cognitive complaints are a major issue in cancer survivors. Many complex mechanisms are involved in these cognitive difficulties, such as direct neurotoxic effects caused by chemotherapy or by indirect effect such as cytokine levels.^17^, ^18^ Nevertheless, the exact mechanisms are not yet well understood and more information is needed to identify subgroups of patients at risk.^18^

In case of combined treatments, the start of hormone therapy can coincide with the end of chemotherapy. Thus, cognitive complaints during hormone therapy may be attributable to the end of chemotherapy. Furthermore, in supplementary analyses Table 1, we observed that the odds of cognitive complaint were lower in patients who had surgery, radiotherapy and hormonotherapy compared to those who went through surgery, radiotherapy and chemotherapy.

In this large survey which included mainly breast cancer survivors, cognitive difficulties were the second troublesome postcancer treatment symptoms after fatigue and before pain. Previous studies about postcancer treatment side effects have shown that cognitive difficulties are often reported in breast cancer survivors, with many survivors asserting that it is their most troublesome posttreatment symptom.^2^ In addition to the impact on quality of life, these difficulties have repercussions on work resumption (39% of our sample, who had cancer–related cognitive complaints and were employed, were concerned with important impacts and 37% by a little impact) and could disturb the ability to work^2^, ^3^, ^4^ if cognitive disorders persist.

Our findings are in line with previous studies which reported a strong association between chemotherapy and cognitive complaints.^7^, ^9^, ^10^, ^19^ Cognitive complaints have also been associated, to a lesser extent, with hormone therapy, as frequently reported.^9^, ^10^, ^20^ Furthermore, participants described a start of cognitive difficulties mainly during chemotherapy and after chemotherapy, and also during hormone therapy.

Self–reported sleep difficulties were also strongly associated with cancer–related cognitive complaints. This is a novel result as most studies on cognitive complaints have not included the assessment of this parameter.^7^, ^8^, ^9^, ^10^, ^11^, ^21^ It is however well established that sleep disturbances are associated with cognition and most particularly in older adults.^22^, ^23^ Moreover, in cancer patients, sleep problems are also associated with these complaints.^24^, ^25^ This highlights the importance of addressing reversible factors associated with cognitive complaints,^26^ such as sleep difficulties.

The present survey is the first to include preexisting knowledge about chemotherapy–associated cognitive problems assessment. Results showed that this knowledge was strongly associated with cancer–related cognitive complaints, which could suggest that preexisting knowledge could increase major cognitive complaints, in line with previous studies in which the influence of informing patients about cognitive impairment postchemotherapy has been assessed.^27^, ^28^ This relationship between preexisting knowledge and cognitive complaints could also be explained by participants’ motivation and ability to access information explaining the posttreatment symptoms they were experiencing once they occurred. Reassuring information about occurrence on chemotherapy side‐effect does not seem to be sufficient to reduce cognitive complaints.^30^ If some recommendations exist on including cognitive difficulties in the list of cancer treatments side effects,^26^ it is important to find a way to inform patients about cognitive side effects without worsening them.^30^ Further studies on cognitive complaints should include the assessment of this preexisting knowledge.

Age was also associated with cancer–related cognitive complaints: older patients had fewer complaints than younger. This finding could be explained by the increase of cognitive complaints with normal aging.^31^ On the other hand, older individual's expectation about their cognition might be declining with age. Older patients who had cognitive complaints did not systematically associate cognitive complaints with cancer treatments.

Other factors such as current anxiety and depression have been associated with current cognitive complaints.^6^, ^7^ Although we did not include assessment of anxiety/depression, we assessed the consumption of psychotropic treatments and found no association.

According to a previous study that showed that exercise frequency was related to cognitive complaints in cancer patients,^32^ we found that the duration of cancer–related cognitive complaints was shorter in participants who practiced more physical activity. Furthermore, the duration of cancer–related cognitive complaints was shorter in participants who were currently on sick leave. This result could be explained by the fact that our population was heterogeneous according to postcurative treatment time. Indeed, some participants could be currently on sick leave because they completed curative treatment short time ago and they may have current cognitive complaints during the survey while others had long–term curative treatments with no current cancer–related cognitive complaints.

Studies on patient expectations for support of cognitive disorders are scarce. Most cancer survivors who participated in this survey and who had cancer–related cognitive complaints would like to receive support (75%) and especially cognitive training, more or less associated with psychological support and physical activity, rather than therapeutic support. These results confirm the strong demand for cognitive difficulties support and the interest in cognitive support reported by small previous studies.^12^, ^13^ Currently, various methods of cognitive difficulties support are being evaluated (cognitive, physical, behavioral and drug intervention^33^). The best current evidence supports cognitive rehabilitation programs which particularly improve cognitive complaints rather than objective cognitive performances.^34^, ^35^ The largest randomized study based on cognitive training (n = 242), reported a reduction of cognitive complaints for cancer survivors who followed a web–based cognitive rehabilitation program compared to those who had standard care.^34^

Recently, in practice, the American Cancer Society/ASCO Breast Cancer Survivorship Care recommended that primary care clinicians should treat optimally cognitive difficulties when possible and refer patients with signs of cognitive impairment for neurocognitive assessment and rehabilitation, including group cognitive training if available.^37^ Breast cancer survivors with cognitive difficulties have reported little discussion with a medical provider about their cognitive symptoms (37%) and among them, only 30% reported receiving treatments to address cognitive symptoms.^9^ Indeed, oncologists are uncertain about the available potential support strategies^38^ and in most cancer centers, cognitive support is not yet included in the portfolio of supportive care.^18^

Study limitations include the online format of this survey with selective participation and a sample not representative of all cancer survivors. They were mainly breast cancer survivors due to initial “*Seintinelles*” platform access only for breast cancer women, with a more recent access to other cancer patients, whatever gender. When we restricted the analyses to breast cancer survivors, we observed similar results. For this reason, the generalizability of the present study may not be applied to other type of cancer survivors. Furthermore, survivors who participate in social network could have a particular profile, notably socio‐demographic: they were all voluntary for the survey, resulting in potential selection bias. Despite the invitation to participate providing brief information indicating the survey to be relevant to all cancer survivors, it may have led to greater participation of patients with cognitive complaints than those without. This could result in overestimation of cognitive complaints among participants. From the perspective of assessing the cognitive complaints experienced (present or past), we used a general question that has not been validated and that is not issued from a validated self–report questionnaire (since the latter only assesses current cognitive complaints), which could induce an overestimation of cognitive complain. Regarding the duration of cognitive complaints in our study, it may be underestimated as some cancer survivors participants may currently suffer from cancer cognitive complaints.

Considering heterogeneous results of cognitive complaints after cancer treatments in previous cohort studies,^8^, ^10^, ^21^ the cross–sectional design of the present study is a limitation and longitudinal studies with a follow up of more than one year should be encouraged. Finally, we did not assess anxiety and depression with a specific question. Nevertheless based on a large web–based survey the present study is a valuable contribution in the field of cancer and cognition.

## 5 CONCLUSIONS

Through a web–based survey including mainly breast cancer survivors, a large proportion of participants reported cognitive complaints, which reinforces that cognitive difficulties are a major issue in cancer survivors. Chemotherapy, self–reported sleep difficulties and preexisting knowledge about chemotherapy–associated cognitive problems were strongly associated with cancer–related cognitive complaints. Most cancer survivors would like to receive support and especially cognitive training. The promising initial results of cognitive rehabilitation programs should be confirmed in the future.

## CONFLICT OF INTEREST

None declared.

## AUTHORS’ CONTRIBUTIONS

Marie Lange: Conceptualization, funding acquisition, investigation, ressources, validation, writing – original draft. Idlir Licaj: formal analysis, writing – original draft. Bénédicte Clarisse: funding acquisition, project administration, writing – review and editing. Xavier Humbert: ressources, writing – review and editing. Jean‐Michel Grellard: project administration. Laure Tron: funding acquisition, ressources, writing – review and editing. Florence Joly: Conceptualization, funding acquisition, ressources, writing – original draft. All authors consented to manuscript submission and publication.

## References

[1] Joly F , Giffard B , Rigal O , et al. Impact of cancer and its treatments on cognitive function: advances in research from the Paris international cognition and cancer task force symposium and update since 2012. J Pain Symptom Manage. 2015;50:830‐841. 26344551 10.1016/j.jpainsymman.2015.06.019

[2] Boykoff N , Moieni M , Subramanian SK . Confronting chemobrain: an in‐depth look at survivors' reports of impact on work, social networks, and health care response. J Cancer Surviv. 2009;3:223‐232. 19760150 10.1007/s11764-009-0098-x PMC2775113

[3] Nieuwenhuijsen K , de Boer A , Spelten E , Sprangers MA , Verbeek JH . The role of neuropsychological functioning in cancer survivors' return to work one year after diagnosis. Psycho‐Oncology. 2009;18:589‐597. 18942672 10.1002/pon.1439

[4] Von Ah D , Habermann B , Carpenter JS , Schneider BL . Impact of perceived cognitive impairment in breast cancer survivors. Eur J Oncol Nurs. 2013;17:236‐241. 22901546 10.1016/j.ejon.2012.06.002

[5] Pullens MJ , De Vries J , Roukema JA . Subjective cognitive dysfunction in breast cancer patients: a systematic review. Psycho‐Oncology. 2010;19:1127‐1138. 20020424 10.1002/pon.1673

[6] O'Farrell E , Smith A , Collins B . Objective‐subjective disparity in cancer‐related cognitive impairment: does the use of change measures help reconcile the difference? Psychooncology. 2016. 10.1002/pon.4190 27278814

[7] Dhillon HM , Tannock IF , Pond GR , Renton C , Rourke SB , Vardy JL . Perceived cognitive impairment in people with colorectal cancer who do and do not receive chemotherapy. J Cancer Surviv. 2017. 10.1007/s11764-017-0656-6 29080061

[8] Mandelblatt JS , Clapp JD , Luta G , et al. Long‐term trajectories of self‐reported cognitive function in a cohort of older survivors of breast cancer: CALGB 369901 (Alliance). Cancer. 2016. 10.1002/cncr.30208 PMC5113662 27447359

[9] Buchanan ND , Dasari S , Rodriguez JL , et al. Post‐treatment neurocognition and psychosocial care among breast cancer survivors. Am J Prev Med. 2015;49:S498‐S508. 26590645 10.1016/j.amepre.2015.08.013 PMC4656130

[10] Schmidt JE , Beckjord E , Bovbjerg DH , et al. Prevalence of perceived cognitive dysfunction in survivors of a wide range of cancers: results from the 2010 LIVESTRONG survey. J Cancer Surviv. 2016;10:302‐311. 26238504 10.1007/s11764-015-0476-5 PMC5772767

[11] Janelsins MC , Heckler CE , Peppone LJ , et al. Cognitive complaints in survivors of breast cancer after chemotherapy compared with age‐matched controls: an analysis from a nationwide, multicenter, prospective longitudinal study. J Clin Oncol. 2017;35:506‐514. 28029304 10.1200/JCO.2016.68.5826 PMC5455314

[12] Munir F , Burrows J , Yarker J , Kalawsky K , Bains M . Women's perceptions of chemotherapy‐induced cognitive side affects on work ability: a focus group study. J Clin Nurs. 2010;19:1362‐1370. 20500346 10.1111/j.1365-2702.2009.03006.x

[13] Le Fel J , Daireaux A , Vandenbosshe S , et al. Impact of cancer treatments on cognitive functions: the patients' view, their expectation and their interest in participating to cognitive rehabilitation workshops. Bull Cancer. 2013;100:223‐229. 23501351 10.1684/bdc.2013.1710

[14] Bauquier C , Pannard M . Preau M . [The Seintinelles: an innovative approach to promoting Community‐Based Research and sustaining health democracy in oncology]. Sante Publique. 2017;29:547‐550. 29034669 10.3917/spub.174.0547

[15] Veierød MB , Lydersen S , Laake P . Medical statistics in clinical and epidemiological research (Gyldendal akademisk edition). Oslo, Norway: Gyldendal; 2012.

[16] da Silva M , Weiderpass E , Licaj I , Rylander C . Factors Associated with High Weight Gain and Obesity Duration: The Norwegian Women and Cancer (NOWAC) Study. Obes Facts. 2018;11:381–392. 10.1159/000492002 30308488 PMC6257091

[17] Janelsins MC , Kohli S , Mohile SG , Usuki K , Ahles TA , Morrow GR . An update on cancer‐ and chemotherapy‐related cognitive dysfunction: current status. Semin Oncol. 2011;38:431‐438. 21600374 10.1053/j.seminoncol.2011.03.014 PMC3120018

[18] Lange M , Joly F . How to identify and manage cognitive dysfunction after breast cancer treatment. J Oncol Pract. 2017;13:784‐790. 29232539 10.1200/JOP.2017.026286

[19] Lange M , Heutte N , Rigal O , et al. Decline in cognitive functions in elderly early‐stage breast cancer patients after adjuvant treatment. Oncologist. 2016;21:1337‐1348. 27473044 10.1634/theoncologist.2016-0014 PMC5189619

[20] Hermelink K , Küchenhoff H , Untch M , et al. Two different sides of 'chemobrain': determinants and nondeterminants of self‐perceived cognitive dysfunction in a prospective, randomized, multicenter study. Psycho‐Oncology. 2010;19:1321‐1328. 20127909 10.1002/pon.1695

[21] Amidi A , Christensen S , Mehlsen M , Jensen AB , Pedersen AD , Zachariae R . Long‐term subjective cognitive functioning following adjuvant systemic treatment: 7–9 years follow‐up of a nationwide cohort of women treated for primary breast cancer. Br J Cancer. 2015;113:794‐801. 26171932 10.1038/bjc.2015.243 PMC4559822

[22] Leng Y , McEvoy CT , Allen IE , Yaffe K . Association of sleep‐disordered breathing with cognitive function and risk of cognitive impairment: a systematic review and meta‐analysis. JAMA Neurol. 2017;74:1237‐1245. 28846764 10.1001/jamaneurol.2017.2180 PMC5710301

[23] Altena E , Ramautar JR , Van Der Werf YD , Van Someren EJ . Do sleep complaints contribute to age‐related cognitive decline? Prog Brain Res. 2010;185:181‐205. 21075240 10.1016/B978-0-444-53702-7.00011-7

[24] Von Ah D , Tallman EF . Perceived cognitive function in breast cancer survivors: evaluating relationships with objective cognitive performance and other symptoms using the functional assessment of cancer therapy‐cognitive function instrument. J Pain Symptom Manage. 2015;49:697‐706. 25240787 10.1016/j.jpainsymman.2014.08.012

[25] Ng T , Dorajoo SR , Cheung YT , et al. Distinct and heterogeneous trajectories of self‐perceived cognitive impairment among Asian breast cancer survivors. Psychooncology. 2018;27:1185‐1192. 29315963 10.1002/pon.4635

[26] Vardy JL , Bray VJ , Dhillon HM . Cancer‐induced cognitive impairment: practical solutions to reduce and manage the challenge. Future Oncol. 2017;13:767‐771. 28266247 10.2217/fon-2017-0027

[27] Schagen SB , Das E , Vermeulen I . Information about chemotherapy‐associated cognitive problems contributes to cognitive problems in cancer patients. Psycho‐Oncology. 2012;21:1132‐1135. 21769988 10.1002/pon.2011

[28] Jacobs SR , Jacobsen PB , Booth‐Jones M , Wagner LI , Anasetti C . Evaluation of the functional assessment of cancer therapy cognitive scale with hematopoietic stem cell transplant patients. J Pain Symptom Manage. 2007;33:13‐23. 17196903 10.1016/j.jpainsymman.2006.06.011

[29] Schagen SB , Das E , van Dam FS . The influence of priming and pre‐existing knowledge of chemotherapy‐associated cognitive complaints on the reporting of such complaints in breast cancer patients. Psycho‐Oncology. 2009;18:674‐678. 19021129 10.1002/pon.1454

[30] Jacobs W , Das E , Schagen SB . Increased cognitive problem reporting after information about chemotherapy‐induced cognitive decline: The moderating role of stigma consciousness. Psychol Health. 2017;32:78‐93. 27701901 10.1080/08870446.2016.1244535

[31] Reid LM , Maclullich AM . Subjective memory complaints and cognitive impairment in older people. Dement Geriatr Cogn Disord. 2006;22:471‐485. 17047326 10.1159/000096295

[32] Myers JS , Wick JA , Klemp J . Potential factors associated with perceived cognitive impairment in breast cancer survivors. Support Care Cancer. 2015;23:3219‐3228. 25832894 10.1007/s00520-015-2708-7 PMC4586297

[33] Chan RJ , McCarthy AL , Devenish J , Sullivan KA , Chan A . Systematic review of pharmacologic and non‐pharmacologic interventions to manage cognitive alterations after chemotherapy for breast cancer. Eur J Cancer. 2015;51:437‐450. 25623439 10.1016/j.ejca.2014.12.017

[34] Bray VJ , Dhillon HM , Bell ML , et al. Evaluation of a web‐based cognitive rehabilitation program in cancer survivors reporting cognitive symptoms after chemotherapy. J Clin Oncol. 2017;35:217‐225. 28056205 10.1200/JCO.2016.67.8201

[35] Ercoli Lm , Petersen L , Hunter Am , et al. Cognitive rehabilitation group intervention for breast cancer survivors: results of a randomized clinical trial. Psychooncology. 2015;24:1360‐1367. 25759235 10.1002/pon.3769

[36] Von Ah D , Carpenter JS , Saykin A , et al. Advanced cognitive training for breast cancer survivors: a randomized controlled trial. Breast Cancer Res Treat. 2012;135:799‐809. 22918524 10.1007/s10549-012-2210-6 PMC3677054

[37] Runowicz CD , Leach CR , Henry NL , et al. American cancer society/American society of clinical oncology breast cancer survivorship care guideline. J Clin Oncol. 2016;34:611‐635. 26644543 10.1200/JCO.2015.64.3809

[38] Smidt K , Mackenzie L , Dhillon H , Vardy J , Lewis J , Loh SY . The perceptions of Australian oncologists about cognitive changes in cancer survivors. Support Care Cancer. 2016;24:4679‐4687. 27320905 10.1007/s00520-016-3315-y

