# Supplementary material for: Cognitive complaints in cancer survivors and expectations for support: Results from a web–based survey
Source: Cancer Med. 2019 Mar 18;8(5):2654–63. doi: 10.1002/cam4.2069 (PMC6536919; doi:10.1002/cam4.2069)
Supplement: Supplementary file 1 [file CAM4-8-2654-s001.docx]

**Supplementary analysis SA Table 1** Multivariable logistic regression OR and 95% CI of combined treatments and Cancer cognitive complaints

| **Main possible treatments combinations** | N cases^¥^ | Multivariable* OR and 95% CI |
| --- | --- | --- |
| Surgery, radiotherapy and chemotherapy | 189 | 1.00 (Reference) |
| Surgery, and radiotherapy | 41 | **0.17 (0.09-0.33)** |
| Surgery, radiotherapy and hormonotherapy | 120 | **0.44 (0.28-0.68)** |
| Surgery, radiotherapy chemotherapy and hormonotherapy | 564 | **1.67 (1.12-2.47)** |
| Surgery, radiotherapy, chemotherapy, hormonotherapy, and target therapy | 65 | 1.19 (0.59-2.37) |
| Surgery, and chemotherapy | 57 | 1.24 (0.60-2.54) |
| Surgery, and targeted therapy | 38 | **0.23 (0.11-0.50)** |
| Other treatments combinations | 140 | 0.84 (0.36-1.32) |

* Multivariable logistic model are adjusted on: Post-cancer curative treatment time, Cancer metastasis, Age (years), Employment status, Self-reported sleep difficulties, Frequency of psychotropic treatments, Pre-existing knowledge of treatment affect in cognition. In bold in the table: significant results.

**SA Table 2.** Multivariable Odds Ratios (OR) and 95% Confidence Intervals (CI) of breast cancer cognitive complaints (n=1 393) and clinical, demographic and lifestyle characteristics, based on chemotherapy model.

|  |  | Cancer cognitive complaints |
| --- | --- | --- |
|  | n  Cases^¥^1066 | Model |
|  |  | Multivariable** |
|  |  | OR (95% CI) |
| Chemotherapy |  |  |
| No | 179 | 1.00 (Reference) |
| Yes | 887 | **3.72 (2.77-5.00)** |
| Post-cancer curative treatment time |  |  |
| ≤ 1 yr | 300 | 1.00 (Reference) |
| 1-3 yrs | 403 | 1.00 (0.69-1.44) |
| ≥3 yrs | 363 | 0.75 (0.52-1.08) |
| Cancer without metastasis |  |  |
| No | 933 | 1.00 (Reference) |
| Yes | 133 | 1.30 (0.80-2.10) |
| Age |  |  |
| 21-64 yrs | 1003 | 1.00 (Reference) |
| ≥65 yrs | 63 | **0.34 (0.20-0.58)** |
| Employment status |  |  |
| Employed (Full-time or part-time) | 618 | 1.00 (Reference) |
| Sick leave | 126 | 1.23 (0.73-2.10) |
| Student, Retired | 1554 | 0.89 (0.57-1.38) |
| Unemployment | 51 | 0.98 (0.50-1.91) |
| Other | 117 | 1.19 (0.73-1.92) |
| Self-reported sleep difficulties |  |  |
| Never | 62 | 1.00 (Reference) |
| Sometimes | 347 | 1.50 (0.96-2.67) |
| Often | 567 | **2.41 (1.47-3.99)** |
| Frequency of psychotropic treatments |  |  |
| Never | 681 | 1.00 (Reference) |
| <1x/month | 134 | 1.26 (0.82-1.95) |
| ≥1x/month and <1x/week | 61 | 1.31 (0.70-2.46) |
| ≥1x/week | 190 | 1.24 (0.84-1.85) |
| Pre-existing knowledge* |  |  |
| No | 463 | 1.00 (Reference) |
| Yes | 603 | **1.67 (1.24-2.24)** |

In bold in the table: significant results.

* Pre-existing knowledge about chemotherapy-associated cognitive problems

** Mutually adjusted

^¥^ Cancer survivors declaring cognitive complaints
